# Hospital News

**Published:** 1923-01

**Authors:** 


### HOSPITAL NEWS.

Since out-patients' payments were started three years ago at King's College Hospital, the sums raised have been £3,884, £5,681 (fee raised to 1s.), and £6,123. The number of attendances and the proportion of those who pay tends to decrease, partly because the scheme for weekly subscribers of 2d. exempts the latter from payments.

The question whether payments received for the treatment of war pension cases should or should not be regarded as extraordinary income has been disturbing the minds of various provincial hospitals seeking a share of the Government grant. The London authorities incline to the view that these payments are extraordinary income, which, therefore, makes the sum to be regarded as "new money" larger and more difficult to raise than it would otherwise be.

The Government Grant to King Edward VII. Hospital, Cardiff, of £6,940 has given great encouragement to that Institution, which has, during the past year, been making steady advance in its financial position. The balance of the Lord Mayor's Fund (1920) has been handed over to the Hospital, with the result that the overdraft, which approached six figures, has been reduced to £36,000. It had been a reproach to the Hospital that the annual subscribers numbered only about 500, but within the past three months an energetic Committee has been formed at the Docks, under the Chairmanship of Mr. T. P. Thomas, J.P., who has worked in close conjunction with Sir William Diamond, the Chairman of the Board of Management. The result so far is that over 300 additional annual subscribers have been added, with an aggregate annual subscription list of over £1,200. At the same time, the Offices at the Docks are being approached, and in many of these systematic collections have already been instituted. It is intended early in the New Year to extend Mr. T. P. Thomas's scheme throughout the whole of the City of Cardiff and the wide outlying districts served by the Hospital. Altogether, 1922 shows promise, in spite of the grave industrial depression, of being one of the most promising years in the history of the Hospital, and the Chairman of the Board of Management, Sir William H. Diamond, is to be warmly congratulated on the results already apparent of his efforts to place the Hospital on a sound financial footing.

Dame Margaret Lloyd George has contributed £10, and Messrs. Baird, Ltd., Flagstaff Quarries, Penmon, Beaumaris, 100 guineas toward the extension fund of the Carnarvonshire and Anglesey Infirmary, Bangor. At least £20,000 is being aimed at to provide additional accommodation, an increased staff and re-equipment.

The past year has seen such a remarkable change for the better in the finances of the Derbyshire Royal Infirmary that the figures deserve quoting. The income increased by £2,700, the expenditure diminished by £4,470, and on the former side was a new item of revenue, namely, £1,010 from certain approved societies. Thus the ordinary income sufficed for the expenditure, and left a balance in hand of £150. Much credit is due to the efforts of the Saturday Fund, which raised a sum of £1,500 more than in previous years. The amount of work done, and the number of patients treated, were also greater, so that from both points of view the turn-over is encouraging, though there still remains a deficit, happily no more than £1,425, from former years. The employers have seconded the efforts of their staffs, in consequence Mr. Walter Banks, the secretary, has probably less anxiety at the moment than any manager of a hospital of similar size.

Despite unemployment the Westmorland and Cumberland Hospital collections again made a record, and have been able to distribute £3,460. The Saturday collections were almost one-third greater than in the previous year.

Miss Riddell, of Belfast, has given £9,000 to the endowment fund of the Belfast Maternity Hospital.

At the annual national conference of Industrial Assurance Approved Societies, Mr. A. C. Thompson, who has been re-elected president, announced that a sum of £175,000 per annum has been allotted by the societies to voluntary hospitals giving treatment to their members.

The Birmingham General Hospital has sold the organ in the Town Hall to the Birmingham Corporation for £10,000, and the committee has decided to waive certain rights of access pertaining to the hospital under the Corporation Consolidated Acts. Mr. D. V. Johnstone, chairman of the Hospital Board, has announced that all the closed beds have been reopened.

The Birmingham Hospital Saturday Fund amounted to £49,053, compared with £58,910 last year. The decline is attributed to unemployment.

The sum of £26,000 raised by the Salford War Memorial Fund has been used to endow a ward with twenty beds in the Salford Royal Hospital.

The East Lancashire Workpeople's Hospital Fund has raised £12,282 in the past year, an increase of £2,518 on the previous total. The scheme was only started in 1918.

An organisation has been at work in Hastings and St. Leonards on behalf of the East Sussex Hospital to remove the deficit of £6,139. Because a fête in its aid was spoilt through bad weather, it was decided to substitute a fancy fair in the Women's Ward block of the new hospital now being built; and in addition, an illuminated carnival was arranged by local philanthropic, social, and working-class organisations. The combined effort is expected to reduce the debt by £5,000' a result upon which Mr. J. Adams, the organiser, has been congratulated.

The Lowestoft Hospital and North Suffolk Contribution Scheme raised £2,515 during the past year.

A shop assistant has complained in the Derby papers that the class to which he belongs is not encouraged to subscribe to hospitals. He urged hospital secretaries not only to have a small collecting box in each shop, but to "place it in charge of the manager or manageress," and let him or her approach the staff. Though shop assistants are but moderately paid, the writer points out that their earnings are regular.

The Walsall General Hospital, like certain other institutions, is feeling in a double sense the loss of the grants from the War Pension Authorities in respect of the treatment of ex-Service men. In 1920 these grants amounted to £2,500, but in the succeeding year to £500 only. But the Voluntary Hospital Commission is making its grants on a basis that includes the income for 1920, which happens to be highest in the hospital's history. We note the appearance in the local papers of descriptive articles upon the hospital's special departments, which should interest local readers and encourage them to help to maintain the expensive standard of this work.

The empty wards of the Royal Hospital, Sheffield, are in process of re-opening, thanks largely to the success of the Hospital Contributors' Council, to which 1,400 firms are affiliated. The committees and medical staffs of the Royal Hospital and the Royal Infirmary have agreed on a plan to exchange patients in order to reduce the long waiting lists. The chairman of the Royal, Mr. Philip K. Wake, expects this to pay its way this year. The income from the 1d. in the £ scheme is already £40,000, and is expected to reach £50,000 next year.

The Birmingham Cripples' Union has moved into new premises near Five Ways, from Daimler House, where the work was cramped. The union in all has three departments; the hospital at Woodlands, and the convalescent home at Forelands, Bromsgrove, are the other two.

The new out-patient department at the Chesterfield Royal Hospital has been opened. The waiting hall now seats 180. The employers have given half the cost, which was £35,000. The land was given by Mr. Eastwood, and Holywell House, also presented by him, has been converted into a home for the nursing and the domestic staff.

Unemployment is so serious in East Anglia that a contributory scheme has been postponed, but it is hoped to prepare it in time for it to be started in 1924, both in town and country.

The Birmingham Royal Orthopædic Hospital hopes to provide a home for its convalescent patients, co-operation to this end between the hospital and others having failed.

The new wing lately added to the Merthyr General Hospital at a cost of £12,000 by Mr. and Mrs. Seymour Berry, has led to the launching of a fund to provide beds.

The East Riding County Council has been asked to grant £43 toward the first year's expenses (£800) of the East and West Ridings Local Voluntary Hospital Committee.

The proposed new St. Paul's Hospital in Endell Street is at a halt for want of funds, though the entrance hall and an ante-room have been built. Till beds are in use, the hospital receives no help from the Combined Hospitals' Appeal Fund.

Mrs. Henry Wood has advocated ultimately a joint hospital scheme (and for the present some mutual arrangement) for the Tunstall and Burslem, Haywood Hospital, districts. The Tunstall War Memorial Committee is considering a scheme for a Cottage Hospital there.

The Yarraw Convalescent Home, Broadstairs, used as a military hospital during the war, has re opened for the children of middle-class parents of limited means. It can accommodate 50 boys and 50 girls from four to fourteen years old. There is a maintenance charge of 10s. a week for those parents able to pay it, and a stay of four weeks is the minimum. Details can be learnt from the Secretary, 116 Victoria Street, S.W.1.

The " new movement " on behalf of the funds of the nine hospitals in the Borough of St. Marylebone is reflected with success in the organisation of each ward in Bexhill to endow a ward in the East Sussex Hospital.

In the spring the building of the Dr. Elsie Inglis Memorial Hospice, connected with the Edinburgh Hospital and Dispensary for Women and Children is expected to be begun at Abbey Hill.

The scheme for a War Memorial Cottage Hospital at Okehampton, Devonshire, has been approved.

The foundation-stone of the Hornsea Cottage War Memorial Hospital, Yorkshire, has been laid. Thirty masonic lodges were represented at the ceremony.

The London Jewish Hospital is allowing medical practitioners to have their private patients treated at the hospital's new X-ray department at a minimum fee of one guinea. The regulations under which this provision is made are being circulated.

Crofton Convalescent Hospital for Women, Aigburth, has been opened by the Lord Mayor of Liverpool. It is controlled by the Liverpool Hospital Saturday Fund.

The West Hartlepool Maternity Home has been opened by the Mayoress. The building was bought by the Corporation for £3,000 in 1921. It possesses 16 beds, a theatre, and reception room. Each doctor can attend his own patients.

The new Grove House Private Hospital, Barrowford, has been opened. It accommodates 17 patients, and admits medical, surgical, and maternity cases. The secretary is Mr. J. W. Kneeshaw, 7 Hargreaves Street, Burnley.

Through a distressing mistake, discovered only a few minutes before the funeral service, an undertaker in the Provinces lately removed the wrong body from the hospital mortuary. The porter failed to check the body taken, because the undertaker was in great haste and declared that he had known the dead man personally. The widow courteously accepted the assistant secretary's explanation, given at a personal call, and kindly exonerated the porter.

